# Recurrent meningococcal meningitis with complement 6 (C6) deficiency: A case report

**DOI:** 10.1097/MD.0000000000020362

**Published:** 2020-05-22

**Authors:** Ji Yun Bae, Ahrong Ham, Hee Jung Choi, Chung-Jong Kim

**Affiliations:** Department of Internal Medicine, Ewha Womans University School of Medicine, Seoul, Republic of Korea.

**Keywords:** C6 deficiency, complement deficiency, meningococcal meningitis, *Neisseria meningitidis*

## Abstract

**Rationale:**

Late complement deficiency increases susceptibility to meningococcal disease and recurrent infections. In Korea, 5 case reports have described meningococcal disease with complement deficiency. However, C6 deficiency has not been described previously.

**Patient concerns:**

A 21-year-old police trainee presented with recurrent meningococcal meningitis. He was housed in communal living quarters until 20 days before the initial symptom onset.

**Diagnosis:**

He was diagnosed with meningococcal meningitis with C6 deficiency.

**Interventions:**

He was treated with intravenous ceftriaxone. An additional dose of quadrivalent meningococcal conjugate vaccine was administered after discharge.

**Outcomes:**

He was discharged without complications.

**Lessons:**

Screening for complement deficiency is necessary in patients with a history of recurrent meningococcal infections to provide appropriate care and prevent recurrent infections.

## 1 Introduction

*Neisseria meningitidis* colonizes the nasopharyngeal mucosa and occasionally invades the bloodstream, which can cause fatal infections, such as septicemia and meningitis. *N meningitidis* is transmitted exclusively between humans either through respiratory secretions or saliva via air-borne respiratory droplets.^[1]^ Therefore, in a communal living setting, such as a military base or college dormitory, young adults are at an increased risk of meningococcal infections.^[2]^ In addition to environmental factors, it is well established that immunosuppression, asplenia, and a deficiency of the terminal complement cascade (factors C5 through C9) are risk factors for meningococcal meningitis.^[3–5]^

To date, 5 studies have reported meningococcal disease with complement deficiency in South Korea.^[6–10]^ However, a C6 deficiency related to meningococcal meningitis has not been reported. Here, we describe a case of recurrent meningococcal meningitis associated with C6 deficiency in a young adult Korean man.

## 2 Case report

A 21-year-old man visited the emergency room complaining of fever and headache that had lasted for 2 days, accompanied by nausea and vomiting. He had a petechial rash on both legs, hands, and the trunk, which appeared the day before the visit. He was a trainee auxiliary policeman and had finished a 4-week training course 20 days before symptom onset. He had been vaccinated with a quadrivalent meningococcal conjugate vaccine (Menveo) before starting communal living. There was no past medical history.

Upon admission, his blood pressure was 108/66 mmHg, heart rate was 99 beats/min, body temperature was 38.0°C, respiratory rate was 20 breaths/min, Glasgow Coma Scale score was 15, and oxygen saturation was 100%. Further physical examination was unremarkable and he showed no signs of meningeal irritation. Upon admission, blood analyses revealed the following: white blood cell (WBC) count, 26,720 /mm^3^ with 93.8% neutrophils; blood platelet count, 166 × 10^9^ /L; prothrombin international normalized ratio (INR), 1.46; C-reactive protein, 17.95 mg/dL; serum creatinine, 1.44 mg/dL; and serum glucose, 117 mg/dL. Cerebrospinal fluid (CSF) analysis revealed the following: WBC, 4550 /mm^3^ with 89% neutrophils; protein, 135 mg/dL; and glucose, 39 mg/dL. Blood cultures revealed *N meningitidis* serogroup B (MenB), and a CSF culture revealed no microorganisms. He was treated with intravenous ceftriaxone (4 g/day) for 9 days and showed a good clinical response.

After 6 months, he revisited the emergency room complaining of fever and headache accompanied by nausea and vomiting. He also reported upper respiratory symptoms, such as cough, coryza, sputum, and sore throat. His blood pressure was 109/70 mmHg; heart rate, 80 beats/min; temperature, 37.6°C; respiratory rate, 24 breaths/min; Glasgow Coma Scale, 15; and oxygen saturation, 100%. Upon admission, a blood analysis revealed the following: WBC, 12,520 /mm^3^ with 94.6% neutrophils; blood platelet count, 185 × 10^9^ /L; INR, 1.16; C-reactive protein, 0.88 mg/dL; and serum glucose, 111 mg/L. A CSF analysis revealed the following: WBC, 30 /mm^3^ with 93% neutrophils; protein, 25 mg/dL; and glucose, 67 mg/dL.

Blood culture revealed *N meningitidis* again; however, it was not identified in the CSF culture. The microorganism isolated in the second event of meningococcemia was identified as serogroup B and ST-44. Due to the recurrent infection, we performed a complement system function test with a radioimmunoassay. The total hemolytic component level of complement was reduced (6.5 U/mL), and C6 was not detected; all other complement components were within the normal range (C3: 68.7 mg/dL, C4: 13.4 mg/dL, C5: 18.0 mg/dL, C7: 8.6 mg/dL, and C9: 23.0 mg/dL). He was treated with another 14-day course of intravenous ceftriaxone, and he was discharged without complications. He again received a quadrivalent meningococcal conjugate vaccine 2 months after discharge.

The patient was informed of the intention to publish a report of his case, and verbal consent was obtained prior to publication.

## 3 Discussion

To our knowledge, this is the first case of confirmed C6 deficiency in Korea in an individual diagnosed with meningococcal meningitis and sepsis. The patient had two risk factors that were highly associated with meningococcal infection: living in a communal setting and a deficiency in the complement cascade.

C6 is a component of the terminal complement cascade that forms the membrane attack complex (MAC), which comprises C5b, C6, C7, C8, and C9. The MAC plays a critical role in the host defense mechanism by penetrating lipid bilayers, such as the outer membrane of gram-negative bacteria or the viral envelope.^[11,12]^ A deficiency in C6 or any other protein component of the MAC predisposes the individual to recurrent neisserial infections, including meningococcal meningitis.^[13]^ Among individuals with a complement deficiency, the incidence of meningococcal disease is 10,000-fold higher than that observed in the general population, and the recurrence rate is also reported to be higher (41% vs 0.34%).^[3,14]^

C6 is a 120-kD single polypeptide chain containing 913 amino acid residues.^[15–17]^ It is encoded by a single-copy gene located on chromosome 5p13, near the *C7* and *C9* genes.^[18,19]^ The *C6* gene comprises 18 exons within an 80-kb span.^[20]^ C6 deficiency is a heritable autosomal recessive trait.^[21]^ To date, nine distinct molecular defects (2 nonsense, 2 splicing, and 5 small deletions) have been identified. These defects were shown to lead to total or partial C6 deficiency and they are listed in the human gene mutation database.^[22]^ A total C6 deficiency, known as a complete C6 deficiency or C6 quantitatively zero (C6Q0), is found in specific populations, such as Western Cape South Africans. In contrast, it is relatively rare in white individuals from Europe and North America.^[23]^ In Asia, C9 deficiency is quite common among Japanese individuals, with a prevalence of 0.04% to 0.10%; in contrast, the prevalence of C6 deficiency is only 0.0027%.^[3,24]^

The prevalence of complement deficiency has not been investigated in Korea. Previously, only 5 cases of meningococcal disease with complement deficiency have been reported in Korea. Among these, 4 cases involved pediatric patients (7–12 years) and only one was a young adult (a military trainee). Unlike previous cases, the patient we described in the present report had an undetectable C6 level; thus, the complement deficiency in our case was appropriately designated C6Q0.

A complement deficiency should be suspected in patients with meningococcal disease with a history of recurrent infection or atypical progress. Patients with complement deficiencies must be identified to provide appropriate care to prevent recurrent meningococcal infections. The risk of recurrence can be reduced with prophylactic antibiotics and vaccination. Furthermore, genetic counseling should be considered in families that are affected because complement deficiencies are heritable autosomal recessive traits.

In the present case, the isolated microorganism was identified as serogroup B, which is not preventable with a quadrivalent meningococcal serogroups A, C, W, Y conjugate vaccine. The Korean government started issuing quadrivalent meningococcal vaccinations to all military trainees in November 2012.^[25]^ This quadrivalent vaccine was also recommended for individuals with terminal complement deficiencies in Europe and other western countries, where the prevalence of MenB is low.^[26]^ However, serogroup B has become an important health concern worldwide; thus, 2 MenB vaccines were recently introduced: Bexsero (GSK, Brentford, UK) and Trumenba (Pfizer, Philadelphia, PA). Both UK and Ireland have introduced the MenB vaccine into all routine infant immunization programs.^[27,28]^ In Korea, there is insufficient data on serogroups in patients with meningococcal disease. Among the 17 cases of meningococcal disease reported in Korea in 2017, serotyping was carried out in nine cases, of which six were identified as serogroup B.^[29]^ Continual epidemiologic studies are necessary to observe changes in the serogroup distribution, and thus estimate the need for a vaccine against MenB.

## 4 Conclusion

We describe the case of a 21-year old police trainee with meningococcal meningitis that had a C6 deficiency. It is necessary to investigate potential complement deficiencies in patients with a history of recurrent meningococcal infections to provide appropriate care. In the future, more epidemiological studies should be conducted to assess the need for introducing MenB vaccines.

## Author contributions

JYB and AH drafted the manuscript. JYB, AH, and CJK participated in data acquisition. CJK generated the experimental results. CJK and HJC designed the study and reviewed the manuscript for intellectual content. All authors approved the final version of the manuscript.

**Conceptualization:** Ji Yun Bae, Hee Jung Choi, Chung-Jong Kim.

**Data curation:** Ji Yun Bae, Ahrong Ham, Chung-Jong Kim.

**Investigation:** Chung-Jong Kim.

**Supervision:** Hee Jung Choi, Chung-Jong Kim.

**Writing – original draft:** Ji Yun Bae, Ahrong Ham.

**Writing – review & editing:** Hee Jung Choi, Chung-Jong Kim.
